# Knowledge of and attitudes toward HIV/AIDS among Iranian women

**DOI:** 10.4178/epih.e2018037

**Published:** 2018-08-03

**Authors:** Ehsan Zarei, Roghayeh Khabiri, Maryam Tajvar, Shirin Nosratnejad

**Affiliations:** 1 Department of Public Health, Faculty of Health, Shahid Beheshti University of Medical Sciences, Tehran, Iran; 2 Tabriz Health Service Management Research Center, Tabriz University of Medical Sciences, Tabriz, Iran; 3 National Institute of Health Research, Tehran University of Medical Sciences, Tehran, Iran; 4 Department of Health Management and Economics, School of Public Health, Tehran University of Medical Science, Tehran, Iran; 5 Department of Health Management, Faculty of Management and Medical Information, Tabriz University of Medical Sciences, Tabriz, Iran

**Keywords:** Knowledge, Attitude, HIV/AIDS, Iran’s Multiple Indicator Demographic and Health Survey, Iran

## Abstract

**OBJECTIVES:**

This study investigated the knowledge of Iranian women about HIV/AIDS and whether they had accepting attitudes towards people living with human immunodeficiency virus (HIV), and sought to identify factors correlated with their knowledge and attitudes.

**METHODS:**

The data analyzed in the present study were taken from Iran’s Multiple Indicator Demographic and Health Survey, a national survey conducted in 2015. In total, 42,630 women aged 15-49 years were identified through multi-stage stratified cluster random sampling and interviewed. Associations of the socio-demographic characteristics of participants with their knowledge and attitudes were examined using multiple logistic regression analysis.

**RESULTS:**

The majority (79.0%) of Iranian women had heard about HIV/AIDS, but only 19.1% had a comprehensive knowledge. In addition, only 15.4% of women had accepting attitudes toward people with HIV. Being older, married, more highly educated, and wealthier were factors associated with having more comprehensive knowledge of HIV/AIDS, and living in urban areas was associated with having more positive attitudes toward people with HIV.

**CONCLUSIONS:**

The relatively poor knowledge of Iranian women and the low prevalence of accepting attitudes toward people living with HIV highlight the need to develop policies and interventions to overcome this issue, which would be a basis for further prevention of HIV/AIDS in Iran.

## INTRODUCTION

Since the first case of acquired immunodeficiency syndrome (AIDS) was announced in the US in the early 1980s, it has spread rapidly around the world, and it continues to be a major global public health problem [1]. According to the World Health Organization (WHO), at the end of 2015, approximately 36.7 million people were living with human immunodeficiency virus (HIV), and 1.1 million people died from HIV-related causes globally in 2015 [2].

In Iran, the first case of AIDS was reported in 1986. Although, the prevalence of HIV/AIDS in Iran is lower than the average of the world and Eastern Mediterranean countries, it has been estimated in recent years that Iran is facing a dramatic rise in HIV infections. According to the estimations of the WHO/Joint United Nations Programme on HIV/AIDS (UNAIDS), the number of people in Iran currently living with HIV has tripled since 2000, increasing from 24,000 cases to 73,000 (95% confidence interval [CI], 50,000 to 130,000) cases in 2015, of which 26,000 were women aged 15 and above [3,4]. While the total number of registered cases of HIV in Iran has gradually decreased since 2004, the number of women cases has steadily increased; the proportion of women among those infected with HIV has increased from only 6% in 2004 to almost 30% in 2013 [3].

Women play a key role in socioeconomic development in the family-oriented context of Iran, as well as in the welfare and health of their family members and in public health; therefore, they should be focused on as part of HIV/AIDS-related interventions in Iran.

Prevention is the main strategy to deal with the AIDS pandemic. Individuals can reduce their risk of HIV infection by limiting their exposure to known risk factors. Inaccurate knowledge and misperceptions are the main barrier to preventing the spread of HIV/AIDS [1,5-7]. However, knowledge alone is not adequate for preventing and limiting HIV infection among women of reproductive age; instead, knowledge must be accompanied by a sufficiently accepting attitude toward engaging in healthy behaviors [8,9]. Characterizing the population’s knowledge of and attitudes toward people with HIV/AIDS is an essential prerequisite to developing effective educational and preventive interventions [1,10]. Previous studies in Iran have reported inconsistent results. Moreover, most of the available studies had a small sample size, were conducted in small settings such as schools or universities, and focused on the population of a particular city or province [11], or were not based on responses to a survey about AIDS. Therefore, their results have limited external validity and are not generalizable to the overall women population of Iran.

In this study, we aimed to fill this gap by investigating the knowledge and attitudes of Iranian women aged 15-49 years regarding HIV/AIDS, based on data from Iran’s Multiple Indicator Demographic and Health Survey (IrMIDHS), a naturally representative survey conducted in 2015. Additionally, socio-demographic correlates of women’s knowledge of and attitudes toward HIV were identified. We hope that this study provides required information for Iranian policy-makers to develop interventions or to improve preventive strategies for combatting HIV/AIDS in Iran.

## MATERIALS AND METHODS

### Data

The data of this study were taken from the IrMIDHS, which was conducted in 2015 by the Statistics Center of Iran (SCI) and the Ministry of Health and Medical Education (MoHME) [12].

### Sampling and data collection

The IrMIDHS was conducting using multi-stage stratified cluster-random sampling. In this sampling technique, a mixed model with random effects was used to account for inter-cluster correlations. Samples were selected using the probability proportionate to size method to ensure that the women enrolled in the study were representative of the entire community. Thus, based on the proportion of households living in each of the 31 provinces of Iran, the number of households was determined, and the estimated sample for the entire country was 34,860 households, overall. The sampling frame was developed using Iran’s 2011 population and housing census data, which included lists of the postal addresses of all ordinary households living in each county. Equal clusters were created, consisting of 10 households each, and were randomly stratified for the study.

In total, 44,921 women aged 15-49 years who were living in sampled households were enrolled and participated in our study. Women were included in the study if: (1) they were at least 15 years of age (married or single), and (2) they provided consent to participate in the study. Women were excluded from the study if they were not able to respond to the questions for any reason, such as cognitive impairments. A trained interviewer carried out face-to-face interviews with the study participants.

### Measurement

A number of validated and tested questionnaires were used in the IrMIDHS, of which one was specifically developed for women aged 15-49 years. The questionnaires of the IrMIDHS were developed based on the Multiple Indicators Cluster Survey. The women’s questionnaire included questions related to participants’ knowledge and attitudes toward people with HIV, as described below [13]:

Three questions on knowledge of HIV/AIDS prevention: (1) identifying 2 ways of preventing HIV transmission (using condoms and limiting sex to one faithful uninfected partner); (2) knowing that a healthy-looking person can have HIV/AIDS; and (3) rejecting 2 of the 3 most common misconceptions about HIV transmission, including, (a) “HIV can be transmitted by mosquito bites,” (b) “HIV can be transmitted by kissing and hand-shaking with someone with HIV/AIDS,” and (c) “HIV can be transmitted by sharing food with someone with HIV/AIDS.”

Four items on accepting attitudes towards people living with HIV: (1) being willing to care for a family member with HIV/AIDS; (2) buying fresh vegetables from a vendor with HIV/AIDS; (3) believing that a female teacher who is HIV-positive but not sick should be allowed to continue teaching in school; and (4) not keeping it a secret that a family member is HIV-positive.

All the questions had binary “yes” and “no” answers. Women who correctly identified 2 ways of preventing HIV infection, knew that a healthy-looking person may have HIV/AIDS, and rejected 2 of the most common misconceptions about HIV transmission were identified as having comprehensive knowledge about HIV/ AIDS prevention. Women with accepting attitudes toward people with HIV were defined as those who answered “yes” to all 4 items assessing attitudes toward people with HIV. A number of covariates, including age, place of residence, educational level, marital status, and wealth index, were taken from the IrMIDHS and used in our study.

### Statistical analysis

Descriptive statistics was used to describe the characteristics of participants, their knowledge of HIV, and their attitudes toward people with HIV. The associations between socio-demographic characteristics (including age, place of residence, educational level, marital status, and wealth index) and knowledge of HIV/AIDS and attitudes towards people with HIV were examined using multivariate logistic regression analysis. Data were analyzed using SAS version 8.2 (SAS Institute Inc., Cary, NC, USA). The results of the logistic regression analysis were reported as odds ratios (ORs) with 95% CIs, and p-values less than 0.05 were considered to indicate statistical significance.

### Ethics considerations

The ethical committees of SCI and the MoHME approved the IrMIDHS.

## RESULTS

A total of 44,921 women were approached, of whom 42,630 participated (response rate, 94.9%). The mean± standard deviation age was 32.8± 5.6 years, and the proportions of participants in different age groups were as follows: 25.8% (n= 11,307) were 15-24 years old, 33.5% (n= 14,658) were 25-34 years old, 28.5% (n= 12,471) were 35-44 years old, and 12.8% (n= 5,359) were 45-49 years old. Furthermore, 64.7% of participants lived in in urban areas, 71.0% were married, and 58.0% had university educations; however, only a minority (7.3%) were wealthy, according to the wealth index.

### Knowledge about HIV/AIDS and associated factors

Almost 79.0% of the participants (Iranian women aged 15-49 years) had heard about HIV/AIDS, but there was geographical disparity in this indicator; the lowest awareness of HIV/AIDS was among participants from Sistan and Baluchistan and South Khorasan Provinces (53.0%), and the highest rate was found among participants from Tehran Province (88.0%).

The percentages of participants with accurate knowledge about HIV prevention for the 5 different questions are presented in Table 1 according to their socio-demographic characteristics. It was found that 58.6% of the women knew that transmission can be prevented by having gender with one faithful uninfected partner, 56.9% knew that the use of a condom can prevent HIV transmission, and 48.2% knew that both of the above methods can prevent HIV transmission. Misconceptions about the transmission of HIV were considerable, as 62.8% of participants thought that HIV could be transmitted by mosquito bites and 45.0% thought that sharing a meal with someone who is infected with HIV could result in transmission. Only 19.1% of women had comprehensive knowledge of HIV, with a difference between those from rural versus urban areas (11.1 vs. 22.2%, respectively). The pattern also slightly varied by marital status (never married, 19.5 vs. ever married, 17.8%), education (higher among educated people), age group (highest among women aged 20-24 years), and wealth index (Table 1).

The findings of the multivariate analyses indicated that place of residence (urban/rural) was not associated with women’s knowledge about HIV (OR, 1.07; 95% CI, 0.98 to 1.16; p= 0.108). However, age was found to be a significant factor associated with knowledge about HIV, as all age groups presented in the table, except the age group of 20-24 years, had a significantly higher proportion of comprehensive knowledge of HIV/AIDS than the reference group (15-19 years old). Married women were more likely to have a comprehensive knowledge of HIV/AIDS than never-married women (OR,1.23; 95% CI, 1.14 to 1.33; p < 0.001). It was also found that higher income, and especially higher education, were associated with a higher likelihood of having comprehensive knowledge about HIV, compared to lower income and education. The associations in all categories were statistically significant (OR of the highest income compared to the lowest income: 1.37; 95% CI, 1.22 to 1.55; p< 0.001; OR of the highest educated people compared to illiterate people: 4.24; 95% CI, 3.72 to 4.48; p< 0.001). Women who had access to mass media, compared to those who did not, were not significantly different in terms of knowledge about HIV. However, computer and internet use showed statistically significant associations with comprehensive knowledge about HIV (Table 2).

### Accepting attitude toward people living with HIV and associated factors

In this study, only 15.4% of study participants reported an accepting attitude for all 4 questions (Table 3). The majority of the respondents (76.9%) indicated that they were willing to take care of an HIV-positive family member in their own home, 58.8% believed that a HIV-positive women teacher should be allowed to continue teaching in a school, 48.1% would buy fresh vegetables and other goods from an HIV-positive vendor, and 41.1% would not want to keep it as a secret if a family member was infected with HIV.

The results of the multiple logistic regression analysis are shown in Table 4. Most of the variables, including age group, marital status, education, and computer and Internet use, were not significantly associated with an accepting attitude towards people with HIV. Only a marginal effect was found for place of residence, as women living in urban areas were more likely to have an accepting attitude than women living in rural areas (OR, 1.18; 95 CI, 1.00 to 1.40; p= 0.048). Moreover, more positive attitudes were found in the wealth category of poorer people compared to the poorest (OR,1.33; 95% CI, 1.06 to 1.68; p= 0.014). Additionally, women who had access to mass media were significantly more likely to have an accepting attitude than those who did not (OR, 1.71; 95% CI, 1.03 to 2.86; p= 0.038).

## DISCUSSION

This study aimed to assess the knowledge and attitudes of women aged 15-49 years in Iran about HIV/AIDS. The results showed that the majority of women (78.5%) had heard about HIV/AIDS, which is consistent with the results of the IrMIDHS-2010 (79.8%) [13]. Only 19.1% of women had comprehensive knowledge of HIV/AIDS, which is comparable to the results of a similar study conducted in Ethiopia (19.3%) [8]. Comprehensive knowledge of HIV/AIDS is an indicator often used to measure awareness of important facts about HIV transmission and prevention. Correct and comprehensive knowledge of HIV transmission and prevention is important to avoid infection [8]. Lack of access to sexual health information could be a reason underlying the low levels of comprehensive knowledge of women about HIV [11]. Gender inequalities and taboos regarding issues of sexuality and sexual health may also contribute to women’s relatively low levels of knowledge [8].

We found a relatively large knowledge gap and extensive misconceptions about HIV/AIDS transmission and prevention in this survey, which is consistent with the results of the IrMIDHS-2010 [13] and a study in India [14]. Most women knew that a healthy-looking person can be HIV-positive, similar to the result of the IrMIDHS-2010 (70%) and a study among young people in Iran [11, 13]. However, misconceptions about transmission were observed among a large proportion of women; such as the belief that HIV can be transmitted by mosquito bites, sharing a meal with an infected person, and shaking hands. Similar misconceptions have been reported in other studies [6,11,15,16]. These findings suggest the need to strengthen educational interventions, especially by health workers for women. Comprehensive knowledge about HIV will support women to make informed choices about practices that may protect them from HIV/AIDS [1].

The highest knowledge level was in the older age groups, in line with the findings of studies in India [14] and Saudi Arabia [10]. This can be attributed to more exposure to sexual health education, such as mandatory premarital trainings on sexual health and HIV, which are regularly practiced in Iran [11]. Married women showed significant differences in comparison to never-married women in terms of their knowledge about HIV/AIDS, which is expected due to their higher potential exposure to infection. This is similar to the result of a study conducted in Congo and Nigeria [17]. Our results showed that the wealth index was significantly associated with comprehensive knowledge of HIV/AIDS among Iranian women. This result is similar to those reported from other parts of the world [8,17,18]. Socioeconomic status is associated with increased exposure to media and more educational achievements, which have a positive impact on HIV/AIDS knowledge [17]. With increasing levels of education, knowledge also increases, so that women with higher levels of education had better knowledge of HIV/AIDS. This result is consistent with other studies [14,16,17] and this can be attributed to access to more information about the disease. These findings indicate the need to provide suitable education for illiterate and semi-literate women regarding HIV/AIDS transmission, which should be considered in health education programs. The difference in knowledge among rural and urban women was not significant; this finding is inconsistent with the results of the IrMIDHS-2010 (urban, 37% vs. rural, 20%; p< 0.05) [13] and other similar studies [8,16,18]. Since women living in urban areas may have greater access to education, mass media, and HIV/AIDS awareness campaigns [8,11], this finding is unexpected and should be further investigated in subsequent studies.

The Iranian media have not been effective in educating people about HIV and other sexually transmitted infections, mostly because these topics are considered socially taboo and too shameful to be discussed in mass media. A lack of access to sexual health information may lead to limited knowledge about HIV.

The results also showed that 15.4% of women had an accepting attitude toward people living with HIV, showing an improvement compared to the proportion of 9.0% in the IrMIDHS-2010 [13]. However, accepting attitudes were still uncommon, and attitudes should be a topic of particular attention in future educational interventions. Similar results have been reported in previous studies in Iran [11], Bolivia [18], Bahrain [6], and the United Arab Emirates [19]. According to UNAIDS, negative attitudes are common all over the world [8], and are one of the main obstacles in the fight against HIV/AIDS [17].

The most common item for which participants reported an accepting attitude was related to taking care of an HIV-positive family member in one’s own home, which can be attributed to the family-oriented culture of Iran Similar results have been reported in Laos [15] and Saudi Arabia [10]. The lowest rate was related to the disclosure of a family member with HIV/AIDS, which reflects the fact that the stigma of HIV/AIDS is still a major issue in our community. Negative attitudes toward buying fresh vegetables from a HIV-positive shopkeeper slightly changed, dropping from 65% in 2010 [13] to 52% in this survey.

Positive attitudes toward people with HIV were more common among women who had access to mass media and lived in urban areas, which can be attributed to greater access to information about HIV/AIDS. Exposure to mass media has been found to be related to less stigmatizing attitudes towards HIV [20,21].

The relatively negative attitudes we found toward people with HIV may have been due to inaccurate knowledge about HIV transmission and prevention [11,22,23], leading to unnecessary concerns about interacting with people with HIV and higher levels of stigma [10]. Negative attitudes toward HIV are an obstacle to the efficient implementation of educational interventions, voluntary counseling, and HIV testing [15,22], while accepting attitudes have been found to be related to willingness to receive HIV testing [11]. Negative attitudes can be modified through effective educational interventions and information about AIDS [24], which could eventually lead to healthy behaviors. Comprehensive knowledge could be a fundamental factor for building accepting attitudes towards people living with HIV.

This study has revealed important findings about women’s knowledge of and attitudes toward HIV/AIDS at the national level; however, it may have some limitations. As with other studies based on self-report data, the quality of the data may have been affected by social desirability bias, and people may not have expressed their real attitudes in face-to-face interviews.

In conclusion, our study revealed that Iranian women’s knowledge about HIV/AIDS and, in particular, their attitudes toward people with HIV were not acceptable, which should be considered as an important concern. Improving the knowledge and attitudes of the public toward HIV/AIDS has been recognized as an important factor in management of the disease. Therefore, educational programs on HIV/AIDS should be strengthened in schools and health centers in accordance with Islamic and Iranian culture to correct the misconceptions found in this study and to encourage accepting attitudes toward people with HIV, particularly among women living in rural areas, women with a low educational level, and in lower socioeconomic strata. Launching awareness campaigns for the public is another way to improve knowledge and attitudes about HIV/AIDS.

## Figures and Tables

**Table 1.** Percentage of participants with knowledge about HIV prevention methods by their socio-demographic characteristics

Variables		Women who knew transmission can be prevented			Women who knew that a healthy-looking person can be HIV-positive	Women who knew HIV cannot be transmitted			Women with comprehensive knowledge
		Having only one faithful uninfected sex partner	Using a condom every time	Knew both methods		Mosquito bites	Kissing and hand-shaking	Sharing food with someone with HIV	
Total		58.6	56.9	48.2	66.2	37.2	63.5	55.0	19.1
Area	Urban	64.7	62.9	53.9	72.7	41.2	70.2	60.7	22.2
	Rural	43.0	41.5	33.4	49.7	26.8	46.3	40.7	11.1
Age (yr)	15-24	60.7	58.8	49.8	68.9	39.0	66.1	57.3	20.2
	25-29	42.7	42.4	35.6	45.9	23.5	43.3	37.9	10.7
Marital status	Ever married	58.7	58.3	50.4	62.3	33.2	60.9	52.5	17.8
	Never married	58.5	56.5	47.5	67.4	38.4	64.3	55.8	19.5
Education	None	4.6	4.7	3.9	4.0	3.5	4.9	3.7	0.7
	Basic	17.2	15.5	11.9	17.1	9.4	17.1	15.1	2.9
	Secondary	46.2	43.2	35.3	56.7	30.7	52.0	42.2	11.6
	Higher	72.8	71.5	61.2	80.9	45.9	78.3	69.0	25.6
Wealth index (quintile)	Q1 (poorest)	71.8	60.6	59.0	72.2	42.3	49.2	48.7	21.7
	Q2 (poorer)	67.4	58.3	52.7	61.9	31.2	75.6	60.9	17.1
	Q3 (middle)	62.9	59.8	51.7	62.7	32.4	64.5	47.7	10.3
	Q4 (richer)	52.7	50.3	41.4	59.3	34.7	64.5	62.2	19.5
	Q5 (richest)	60.4	59.7	52.2	51.5	37.8	61.8	50.7	21.0

HIV, human immunodeficiency virus.

**Table 2.** Multiple logistic regression analysis of associations between comprehensive knowledge about HIV/AIDS among women and their socio-demographic characteristics

Variables	OR (95% CI)	p-value	Marginal effect
Urban/rural	1.07 (0.98, 1.16)	0.11	0.01
Age (yr)			
15-19	1.00 (reference)		Reference
20-24	1.02 (0.90, 1.16)	0.73	0.00
25-29	1.31 (1.15, 1.48)	<0.001	0.06
30-34	1.45 (1.28, 1.65)	<0.001	0.08
35-39	1.54 (1.34, 1.77)	<0.001	0.10
40-44	1.44 (1.24, 1.67)	<0.001	0.08
45-49	1.52 (1.27, 1.78)	<0.001	0.09
Married/never married	1.23 (1.14, 1.33)	<0.001	0.05
Wealth index (quintile)			
Q1 (poorest)	1.00 (reference)		Reference
Q2 (poorer)	1.18 (1.05, 1.32)	0.005	0.04
Q3 (middle)	1.19 (1.06, 1.34)	0.002	0.04
Q4 (richer)	1.26 (1.12, 1.42)	<0.001	0.05
Q5 (richest)	1.37 (1.22, 1.55)	<0.001	0.07
Education			
Illiterate	1.00 (reference)		Reference
Middle school	1.84 (1.64, 2.07)	<0.001	0.13
Diploma	2.88 (2.56, 3.24)	<0.001	0.24
University degree	4.24 (3.72, 4.48)	<0.001	0.33
Mass media	1.13 (0.90, 1.42)	0.29	0.03
Computer use	1.17 (1.06, 1.28)	0.001	0.03
Internet use	1.43 (1.31, 1.58)	<0.001	0.08

OR, odds ratio; CI, confidence interval.

**Table 3.** Percentage of accepting attitudes toward people with HIV according to the socio-demographic characteristics

Characteristics	Are willing to care for a family member with AIDS in own home	Would buy fresh veg- etables from a shopkeeper or vendor who is HIV-positive	Believe that a woman teacher who is HIV-posi- tive and is not sick should be allowed to continue teaching	Would not want to keep secret that a family member is HIV-positive	Express accepting attitudes on all 4 indicators
Total	76.9	48.1	58.8	41.1	15.4
Area					
Urban	77.6	48.9	60.3	39.8	15.5
Rural	74.5	45.5	53.6	45.6	15.2
Age (yr)					
15-24	77.1	48.4	59.1	41.1	15.6
25-29	75.1	45.1	55.3	41.5	13.6
Marital status					
Ever married	77.9	47.9	58.9	44.3	15.6
Never married	76.6	48.2	58.8	40.2	15.4
Education					
None	55.3	22.1	20.7	38.9	5.4
Basic	71.0	35.0	43.0	50.2	14.0
Secondary	72.2	39.0	51.2	42.8	11.8
Higher	78.8	51.7	62.1	40.2	16.7
Wealth index (quintile)					
Q1 (poorest)	80.9	62.1	74.3	28.9	7.1
Q2 (poorer)	64.2	34.0	56.7	38.1	11.2
Q3 (middle)	70.8	47.4	52.7	44.3	17.0
Q4 (richer)	75.6	50.3	60.0	59.0	19.9
Q5 (richest)	75.7	61.2	64.6	46.7	31.3

HIV, human immunodeficiency virus; AIDS, acquired immunodeficiency syndrome.

**Table 4.** Multiple logistic regression analysis of associations between women’s accepting attitudes towards people with HIV and their socio-demographic characteristics

Variables	OR (95% CI)	p-value	Marginal effect
Urban/rural	1.18 (1.00, 1.40)	0.05	0.04
Age (yr)			
15-19	1.00 (reference)		Reference
20-24	1.05 (0.79, 1.39)	0.74	0.01
25-29	1.03 (0.77, 1.38)	0.83	0.01
30-34	1.23 (0.92, 1.66)	0.16	0.05
35-39	1.06 (0.78, 1.44)	0.70	0.01
40-44	1.11 (0.80, 1.53)	0.54	0.02
45-49	1.25 (0.89, 1.76)	0.19	0.05
Married/never married	1.09 (0.91, 1.30)	0.35	0.02
Wealth index (quintile)			
Q1 (poorest)	1.00 (reference)		Reference
Q2 (poorer)	1.33 (1.06, 1.68)	0.01	0.07
Q3 (middle)	1.18 (0.93, 1.49)	0.16	0.04
Q4 (richer)	1.12 (0.89, 1.42)	0.32	0.03
Q5 (richest)	0.96 (0.74, 1.25)	0.78	-0.01
Education			
Illiterate	1.00 (reference)		Reference
Middle school	1.07 (0.86, 1.31)	0.55	0.01
Diploma	1.08 (0.86, 1.36)	0.50	0.02
University degree	0.88 (0.67, 1.15)	0.35	-0.03
Mass media	1.71 (1.03, 2.86)	0.04	0.13
Computer use	0.99 (0.79, 1.25)	0.97	-0.001
Internet use	1.06 (0.84, 1.35)	0.60	0.01

HIV, human immunodeficiency virus; OR, odds ratio; CI, confidence interval.
